# Production of the short peptide surfactant DAMP4 from glucose or sucrose in high cell density cultures of *Escherichia coli* BL21(DE3)

**DOI:** 10.1186/s12934-014-0099-y

**Published:** 2014-08-19

**Authors:** Michele Bruschi, Jens O Krömer, Jennifer A Steen, Lars K Nielsen

**Affiliations:** Australian Institute for Bioengineering and Nanotechnology (AIBN), cnr Coopers and College Rd (Bldg 75), The University of Queensland, St. Lucia, QLD 4072 Australia; Centre for Microbial Electrosynthesis (CEMES), Advanced Water Management Centre (AWMC), Research Road (Bldg 60), The University of Queensland, St. Lucia, QLD 4072 Australia

**Keywords:** Peptide production, *E. coli*, Glucose, Sucrose, DAMP4

## Abstract

**Background:**

Peptides are increasingly used in industry as highly functional materials. Bacterial production of recombinant peptides has the potential to provide large amounts of renewable and low cost peptides, however, achieving high product titers from Chemically Defined Media (CDM) supplemented with simple sugars remains challenging.

**Results:**

In this work, the short peptide surfactant, DAMP4, was used as a model peptide to investigate production in *Escherichia coli* BL21(DE3), a classical strain used for protein production. Under the same fermentation conditions, switching production of DAMP4 from rich complex media to CDM resulted in a reduction in yield that could be attributed to the reduction in final cell density more so than a significant reduction in specific productivity. To maximize product titer, cell density at induction was maximized using a fed-batch approach. In fed-batch DAMP4 product titer increased 9-fold compared to batch, while maintaining 60% specific productivity. Under the fed-batch conditions, the final product titer of DAMP4 reached more than 7 g/L which is the highest titer of DAMP4 reported to date. To investigate production from sucrose, sucrose metabolism was engineered into BL21(DE3) using a simple plasmid approach. Using this strain, growth and DAMP4 production characteristics obtained from CDM supplemented with sucrose were similar to those obtained when culturing the parent strain on CDM supplemented with glucose.

**Conclusions:**

Production of a model peptide was increased to several grams per liter using a CDM medium with either glucose or sucrose feedstock. It is hoped that this work will contribute cost reduction for production of designer peptide surfactants to facilitate their commercial application.

**Electronic supplementary material:**

The online version of this article (doi:10.1186/s12934-014-0099-y) contains supplementary material, which is available to authorized users.

## Introduction

Peptides are routinely produced by solid-phase chemistry for high-value, fine chemicals applications such as pharmaceuticals [1–4]. As fine chemicals, peptides are assumed to be expensive to produce [5]. However, microbial cells are naturally competent for making and polymerizing amino acids and designer peptides can be recovered by simple cell lysis. Synthetic ‘designer’ peptides can be considered highly programmable polymers that can be precisely assembled from monomers (amino acids) and have applications as highly functional materials with innovative properties. Factors influencing the final cost of recombinant products include the price of feedstock, specific productivity and product titer, and downstream processing costs and efficiency [6].

Short Peptide Surfactants (SPS) are a class of industrial surfactants designed with self-assembly and stimuli-responsive properties [7,8] that could potentially replace traditional petrochemical surfactants such as those used as food additives, detergents and environmental applications [9–12], if they could be produced at low cost [7,10]. The model SPS, DAMP4, is the product of numerous studies [10,13–17]. Expression of this SPS as four tandem repeats of the functional unit [16] was found to increase yield to ~40% of the Total Cell Proteins (TCP) [15]. Equally, simple thermal treatments and/or salting-out techniques have been developed to recover the product, greatly reducing downstream processing costs [18]. Despite attempts to improve biological peptide production, by optimizing growth medium composition [19] and induction conditions [20], current product titers have not surpassed a few hundred mg/L [17].

Industrial production of low cost commodities in Chemically Defined Media (CDM) with simple sugars as carbon feedstock may lower specific productivity but it provides greater process control and increased process reproducibility [21], and it reduces peptide contamination [22] when compared to complex media alternatives. Typically, glucose would be used as the carbon feedstock, however, sucrose from sugarcane is an ideal feedstock for industrial microbial production as it is inexpensive to refine [23] and has impressive environmental credentials [24]. Despite these advantages, sucrose is not widely used as a feedstock for *E. coli* as the majority of industrial *E. coli* strains do not naturally metabolize sucrose. But recent advances in the understanding of sucrose metabolism in *E. coli* and subsequent strain engineering have created strains that can grow on sucrose at the same rate as they do on glucose [25]; as well as strains that produce 1,4-BDO [26], carotenoids [27], or succinic acid [28]. To date, however, no strain has been engineered to produce peptides or proteins from sucrose, nor has that production been scaled up to high cell density with sucrose as the sole carbon source in engineered *E. coli*.

In this study, *E. coli* BL21 was engineered to metabolize sucrose and used in the production of the SPS DAMP4. DAMP4 production was characterized on rich complex media as well as glucose and sucrose based minimal media in batch and fed-batch fermentation systems. To the best of our knowledge, this is the first report to describe production of a model SPS in high cell density cultures using an engineered strain to metabolize sucrose.

## Results and discussion

### Batch bioreactor complex medium

#### DAMP4 production in complex media

**Figure 1:**
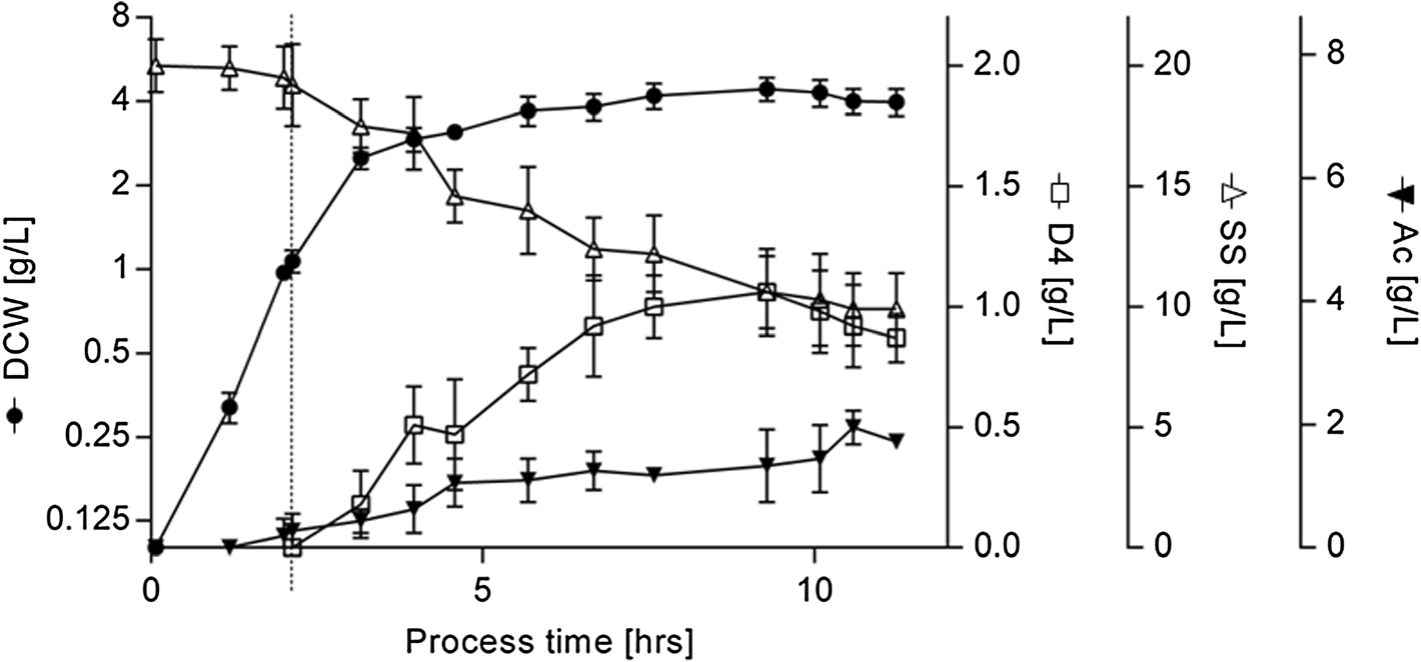
**Growth curves and product formation profile for batch cultivation in LB medium supplemented with glucose (LBG) and induced at DCW ~1g/L.** Biomass formation (*DCW*, circles) is reported on a logarithmic scale; product accumulation (*DAMP4*, open squares), sugar concentration (SS, open triangles) and acetate formation (Ac, inverted filled triangles) are shown. The point of induction is indicated by a dashed vertical bar. Experimental error represented as SD, n ≥ 2.

To establish a reference for cell growth and DAMP4 titer, *E. coli* BL21(DE3) pEDA was grown at low cell density in a rich complex medium consisting of LB medium supplemented with glucose (LBG). To ensure that nutrient availability and by-product accumulation would not limit product formation, induction was performed at 1 g/L dry cell weight (DCW). Under these conditions, cells grew exponentially for ~2 h at which time they were induced. Following induction, the cells continued to grow at a decreasing rate (Figure 1). This cultivation method yielded ~4.3 g/L DCW and ~1 g/L of recombinant DAMP4, corresponding to a maximum yield of ~41% DAMP4 over total cell protein (DAMP4/TCP) in ~6 h expression. Product accumulation was not observed during stationary phase, however after 9 h, a decrease in product yield consistent with loss of cell density was observed.

### Batch bioreactor CDM with glucose or sucrose substrates

#### DAMP4 production in a chemically defined minimal media (CDM) using glucose feedstock

The use of CDM in industrial processes can reduce media cost, maximize substrate conversion into product and minimize interference of media components with the purification of the peptidic product of interest. To investigate the effect of CDM on the production of DAMP4, the batch fermentations were repeated using CDM supplemented with glucose (CDM_G_) (Additional file 1: Figure S1). As expected, switching from rich complex media to CDM reduced growth rate and final biomass concentration. This reduction in CDM was attributed to the cells’ need to divert carbon from fuelling reactions and precursor generation to meet the requirements of complete anabolic metabolism [29,30]. Following induction at 1 g/L DCW, biomass peaked at 2.5 g/L DCW, down from 4 g/L DCW observed for LBG cultures. Production of DAMP4 was also effected; total yield of DAMP4 was reduced from ~1 g/L in LBG to ~0.5 g/L in CDM_G_, corresponding to a maximum yield of ~36% DAMP4/TCP in ~4 h expression.

#### Engineering sucrose metabolism in E. coli BL21 (DE3)

As previously established, production of DAMP4 from sucrose would have several advantages over production from glucose, however, most *E. coli* production strains do not naturally metabolize sucrose. To allow production of DAMP4 from sucrose, the host strain *E. coli* BL21(DE3) pEDA was engineered to metabolize sucrose by co-transforming it with the plasmid p15aCSCx which encodes the *cscAKB* operon from *E. coli* W [31]*.* Provision of sucrose in the media as the sole carbon source was sufficient for plasmid maintenance. Culturing the engineered strain in a bioreactor confirmed that growth rate and biomass yield in sucrose was unchanged when compared to the parent strain in glucose (data not shown). This is in stark contrast to previously published examples of sucrose engineering in *E. coli* in which strains were shown to have unstable sucrose utilization phenotypes, as observed in *E. coli* B [25] and W3110 [32] or demonstrated a reduced growth rate on sucrose when compared to glucose as observed in engineered *E. coli* K-12 strains [27,28,33].

Production of DAMP4 was also repeated using CDM supplemented with sucrose (CDM_S_) using 1 g/L DCW as the point of induction (Additional file 1: Figure S1). The production characteristics (growth rate, biomass and DAMP4 accumulation) on CDM supplemented with sucrose were similar to those obtained from cells cultured on CDM supplemented with glucose (Additional file 1: Figure S1, compare left and right panels).

#### Optimization of production – role of cell density at induction

**Figure 2:**
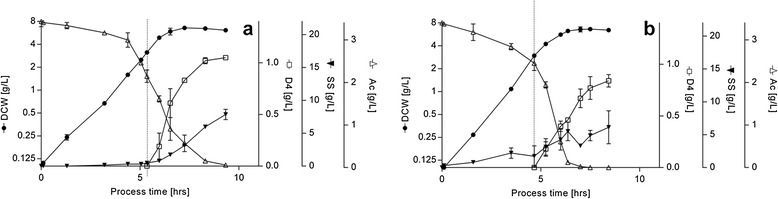
**Growth profiles, product accumulation and extracellular metabolite profiles for batch cultivation in chemically defined medium (CDM) with (a) glucose (CDM**_**G**_ **) or (b) sucrose (CDM**_**S**_ **) as sole carbon source for induction performed at DCW**_**m**_ **.** Biomass formation (*DCW*, circles) is reported on a logarithmic scale; product accumulation (*DAMP4*, open squares), substrate concentration (*SS*, open triangles) and acetate accumulation (*Ac*, inverted filled triangles) are shown. The point of induction is indicated by a dashed vertical bar. Experimental error represented as SD, n ≥ 2.

Upon closer inspection, the reduction in yield observed between complex medium and CDM based fermentations induced at 1 g/L DCW could be attributed to a significant reduction in final cell density rather than a large reduction in specific productivity. To determine the effect of cell density and substrate availability at induction on product accumulation, exponentially growing cells cultured on either glucose or sucrose media were induced at low, mid or high cell density (DCW_l_ ~1 g/L; DCW_m_ ~ 3 g/L; DCW_h_ ~ 6 g/L, respectively, Figure 2 and Table 1, Additional file 1: Figure S1 and Additional file 2: Table S1). The highest product titer was reached inducing at mid cell density (3 g/L DCW_m_ at induction, 6.3 g/L DCW, ~1 g/L DAMP4 corresponding to ~33% DAMP4/DCW). Under these induction conditions, all the substrate provided in the batch phase was consumed and specific productivity was high. Conversely, when inducing at DCW_l_, the excess of substrate available did not provide any more product accumulation after 4h of cultivation (Figure 2 and Additional file 1: Figure S1), which points towards the impact of protein overexpression on cell viability [34], or the limit of other cell functions for longer accumulation of the recombinant product [35]. Inducing at DCW_h_ prompted a decrease in specific productivity, presumably due to insufficient substrate availability. The latter example highlights a characteristic of complex medium in which production following late induction would still be supported by complex nutrients present in the broth [20]. In addition, the higher specific productivity achieved in complex medium compared to CDM shows that *de-novo* amino acid synthesis for peptide production poses a significant burden on the system.

**Table 1:** **Yields of recombinant DAMP4 obtained using either Sucrose or Glucose substrates and induced at DCW**_**m**_ **during batch cultivation or induced at DCW**_**i**_ **with F**_**f**_ **feeding regimen for fed-batch cultivation**

	**Substrate**	**Induction point, Feeding rate**	**Y**_**D4/DCW**_	**Y**_**D4/ss**_ **[%]**	
		**[g/L, mL/h]**	**[mg/g]**	**Total**	**Production**
**▪ Batch**					
G_m_	Glucose	3, NA	171.18 ± 6.37	6.7 ± 0.7	10.4 ± 0.8
S_m_	Sucrose	3, NA	126.44 ± 7.64	6.1 ± 0.4	8.6 ± 0.9
**▪ Fed-Batch**					
G_f_	Glucose	40, 5.5	141.46 ± 10.75	5.4 ± 0.3	9.8 ± 1.6
S_f_	Sucrose	40, 5.5	114.94 ± 15.66	4.3 ± 0.2	7.1 ± 0.2

*Y*_*D4/DCW,*_ specific productivity; *Y*_*D4/ss,*_ substrate conversion into product; *Total,* yield of D4 for the whole process; *Production, yield of D4 during* production phase only. Data set for all conditions are reported in additional material (Additional file 3: Table S1). Experimental error represented as SD, n ≥ 2.

Induction at 3 g/L DCW of CDM supplemented with either sugar achieved a final product concentration of about half of that obtained using complex medium (Figures 1 & 2). Nevertheless, the DAMP4 production rates during batch processes were similar in LBG and CDM_G_; with specific productivity only reduced by ~16% (Table 2). For the commercial production of recombinant peptides, such losses could be acceptable assuming the use of CDM would aid downstream processes and that CDM is cheaper than the complex medium alternative [36] and less prone to batch to batch variations [21]. Moreover, these results demonstrated comparable product accumulation on sucrose compared to glucose across a range of conditions, which distinguishes it from strains that previously showed poorer performance on the former sugar, such as *E. coli* K-12 [27], EC3132 [37] and W3110 [32]. As such, the p15aCSCx plasmid presented here is a significant improvement for the production of peptides from sucrose.

**Table 2:** Rates of consumption/production for main cultivation parameters in Batch or Fed-batch cultivation (expressed as [mmol-C/gDCW*h] unless otherwise stated)

**Name**	**Substrate**	**Phase**	**SUR**	**DCW**	**CO**_**2**_	**Ac**	**Fo**	**D4**	**Balance [%]**
**▪ Batch**									
LBG	LB + glucose	G	48.3 ± 1.3	27.5 ± 3.6	23.5 ± 2.2	ND	ND	0.0 ± 0	ND
		P	23.2 ± 1.1	16.3 ± 2.8	15.9 ± 3.1	3.6 ± 0.3	ND	2.6 ± 0.3	ND
G_m_	Glucose	G	50.2 ± 0.9	24.2 ± 0.3	23.8 ± 2.1	0.6 ± 0.0	1.9 ± 0.1	0.0 ± 0.0	99.3
		P	25.2 ± 0.6	8.9 ± 0.4	13.2 ± 0.5	1.9 ± 0.2	0.0 ± 0.0	2.2 ± 0.1	96.1
S_m_	Sucrose	G	54.6 ± 0.6	24.3 ± 0.5	23.6 ± 0.1	1.9 ± 0.5	0.4 ± 0.1	0.0 ± 0.0	108.9
		P	28.2 ± 0.4	10.0 ± 1.5	14.6 ± 0.3	2.9 ± 0.1	0.6 ± 0.1	2.1 ± 0.1	93.2
**▪ Fed Batch**									
G_f_	Glucose	G	54.3 ± 0.8	23.3 ± 0.3	24.1 ± 1.4	0.6 ± 0.1	2.0 ± 0.4	0.0 ± 0.0	108.7
		F	22.5 ± 0.9	9.8 ± 0.2	11.0 ± 0.6	0.0 ± 0.0	0.1 ± 0.0	0.0 ± 0.0	107.5
		P	4.0 ± 0.2	1.2 ± 0.1	2.0 ± 0.3	0.1 ± 0.0	0.0 ± 0.0	0.4 ± 0.0	107.5
S_f_	Sucrose	G	57.9 ± 1.5	25.1 ± 0.3	25.6 ± 1.1	0.8 ± 0.2	2.6 ± 0.2	0.0 ± 0.0	106.8
		F	24.9 ± 0.6	11.1 ± 0.1	13.2 ± 0.2	0.0 ± 0.0	0.1 ± 0.0	0.0 ± 0.0	102.3
		P	4.6 ± 0.5	1.3 ± 0.4	2.7 ± 0.6	0.1 ± 0.0	0.0 ± 0.0	0.4 ± 0.0	104.1

*Phase* connotes batch Growth phase (*G*), growth during Feeding (*F =* 5.5 mL/h for fed-batch) or Production (*P*) phases (the period between induction and the peak product accumulation); *GR*, Growth Rate; *SUR*, Substrate Uptake Rate; *CO*_*2*_, carbon dioxide; *Ac*, acetate; *Fo,* formate; D4, recombinant DAMP4; *Balance,* percentage ratio between substrate consumption and product formation rates. ND: Not Determined. Experimental error expressed as SD, n ≥ 2.

#### By-product formation

By-product formation directs valuable resources away from the production of the recombinant peptide of interest. During batch cultivation acetate accumulation was observed after induction, reaching ~1.2 g/L maximum (Figure 2), which is consistent with previous observations [35,38]. The shift to acetate production following induction could be partly attributed to the over-representation of certain amino acids (namely Arginine and Methionine) in DAMP4. The amino acid composition of DAMP4 is such that up to 13 mol acetate could be formed per mol DAMP4 as by-product of the production of methionine and arginine. Specifically methionine biosynthesis from a cysteine precursor produced by the cysteine synthase complex (EC 2.5.1.47) results in the production of 1 mol acetate per mol of methionine. In the arginine biosynthesis, the formation of ornithine through the acetyl-ornithine deacetylase (EC 3.1.5.16) yields 1 mol acetate per mol of Arginine. Alternatively, acetate formation could be the product of additional ATP generation required to support recombinant protein formation [39,40] or due to an increased flux around the pyruvate node [41,42].

#### Cultivation and product formation on chemically defined medium fed-batch

**Figure 3:**
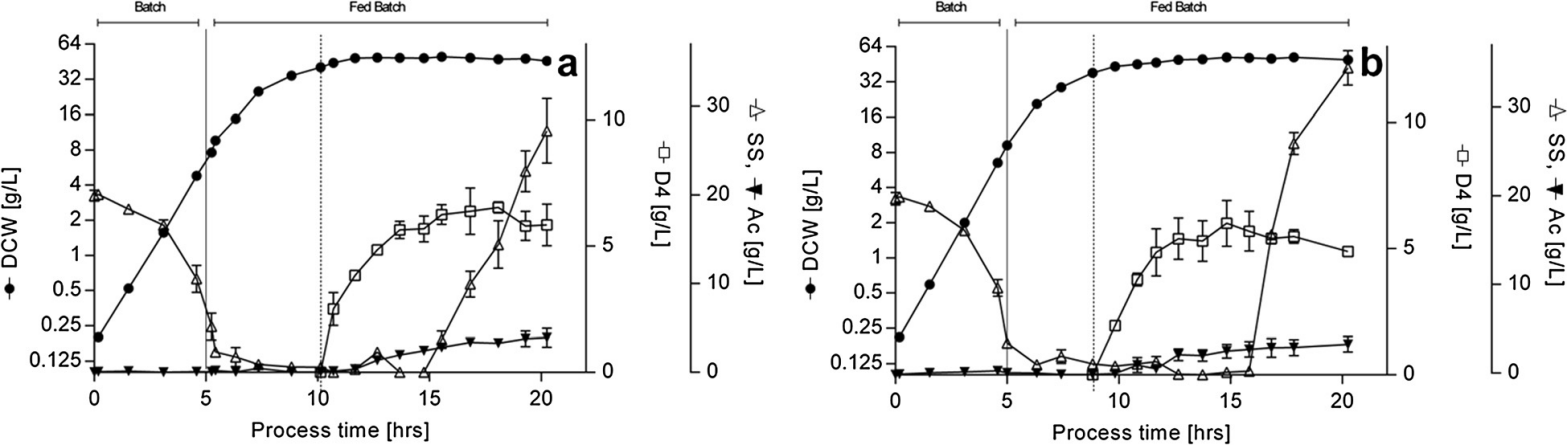
**Growth profiles, product accumulation and extracellular metabolite profiles for fed-batch cultivation in chemically defined medium with (a) glucose (CDM**_**G**_ **) or (b) sucrose (CDM**_**S**_ **) as sole carbon source for induction performed at DCW**_**i**_ **~40 g/L as indicated by the dashed vertical bar and F**_**f**_ **feeding regimen.** Biomass formation (*DCW*, circles), product accumulation (*DAMP4*, open squares) is reported on a logarithmic scale; substrate concentration (*SS*, open triangles) and acetate accumulation (*Ac*, inverted filled triangles) are shown. Experimental error represented as SD, n ≥ 2.

It was proposed that further increase in product titer could be achieved by performing high cell density cultures (HCDC) (reviewed in [43]). Data indicated in cultures where substrate was sufficient, specific productivity remained constant at increasing cell densities, suggesting that further increases in product accumulation could be achieved in higher cell density cultures grown under appropriate conditions. As such, substrate limited fed-batch cultivations with either glucose or sucrose substrates were carried out to increase cell density for induction beyond that of batch cultivation and to supply cells with enough substrate for product generation while limiting overflowing metabolism by maintaining growth rate during feeding below the critical value for acetate accumulation [44,45]. A single pulse IPTG injection performed at ~40 g/L DCW for all cultures triggered the formation of DAMP4. Fed-batch cultivation started as a batch and constant feeding was commenced just before the complete exhaustion of the initially supplied sugar (corresponding to DCW ~9 g/L, ~5 h process time). An initial growth rate at induction of ~0.15 h^−1^ (F_f_ = 5.5 mL/h) was selected in order to limit the effect of acetate formation that may occur at higher growth rates while retaining a relatively high ribosomal concentration [35]. In a second condition growth rate at induction was set at ~0.08 h^−1^ (F_s_ = 4.6 mL/h) to investigate whether growth rate at induction would influence DAMP4 production. During the initial batch phase cultures grew exponentially at their maximum rate in all substrates; after feeding started, cells progressively decreased their growth rate, confirming that growth was carbon controlled (Figure 3). Cells stopped growing about 5 h after induction (Figure 3) as observed for batch experiments. Feeding regimen F_f_ resulted in the highest product titer (7.4 g/L and 6.7 g/L of DAMP4 on glucose and sucrose respectively, corresponding to ~26% and ~21% DAMP4/TCP) (Figure 3 and Additional file 2: Table S1 and S2). Reduction of metabolic activity, as indicated by the accumulation of carbon in the media, was observed after ~15 h of cultivation (Figure 3 and Additional file 3: Figure S2) and could be attributed to either nutrient limitation or metabolic stall induced by recombinant overexpression [34,46,47]. Final cell density reached more than 50 g/L DCW for all cultures, with a maximum of ~55 g/L DCW on sucrose (Additional file 3: Figure S2) in about 15 h of cultivation; acetate production increased abruptly after induction, reaching a maximum of ~5 g/L. Feeding substrate at lower rate (F_s_) led to lower product accumulation, which reached a maximum of ~4.7 g/L (~18% DAMP4/TCP) on sucrose (Additional file 3: Figure S2).

Growth rate at induction can influence product accumulation (reviewed in [48]). In the case of DAMP4, maximum product formation was achieved when induction was performed with cells growing at μ_max_. Presented data clearly showed the dependence of product titer on feeding rate, which is known to influence cells’ growth rate and physiology [49], energy and precursors generation [35], plasmid stability [50], etc. and in turn it might affect protein accumulation [48]. In addition, the high stress of high density cultures may significantly contribute to the decrease in specific productivity.

#### Maximum carbon yield and highest production rate of DAMP4

The performance of the two sugar substrates were compared using carbon moles, with the carbon balance for each condition closed to an average of 102.8 ± 5.3% (Table 2, and Additional file 2: Table S2). DAMP4 yield on batch cultures was ~6% for the whole process and a maximum of ~12% for the sole production phase on both substrates; on fed-batch processes, glucose cultures at fast feeding performed best reaching a product yield of more than 5% for the whole process and ~10% for the production phase (Table 1). DAMP4 accumulation rates varied considerably between batch and fed-batch cultures, owing to the different growth rate for the two-cultivation modes. Indeed, the highest observed DAMP4 production rate in batch cultures was ~2.4 mmol-C_DAMP4_/(g_DCW_*h), while in fed-batch this was ~0.4 mmol-C_DAMP4_/(g_DCW_*h) during production at fast feeding (Table 2). After induction growth rate decreased whereas CO_2_ and acetate production rates increased compared to un-induced growth. Overall, the cultivation process converted most of the substrates into CO_2_ (~65%) and biomass (~30%), with DAMP4 taking maximum ~6% of the substrate. HCDC on glucose had the lowest reduction in product formation compared to batch cultivations. The complete dataset for carbon distribution into products is available as additional material (Additional file 2: Table S1).

The substrate limited fed-batch cultivation, feeding carbon at constant rate, proved to be a simple and effective technique to grow cells to high density and to contain overflow metabolism as demonstrated by the absence of acetate accumulation during the feed phases prior to induction. *E. coli* BL21(DE3) grew up to ~50 g/L DCW in minimal medium fed only with carbon and nitrogen sources and MgSO_4_. Comparing batch cultivation (G_m_, S_m_) with fed-batch cultivation (G_f_, S_f_), final cell density and product titer increased ~8-fold and ~6-fold, respectively, with DAMP4/TCP decreasing of ~0.7-fold (Figures 2 and 3), but the DAMP4 production rate was only about 15% of that reported for the batch phase (Table 2). Growth rate decreased constantly as cell concentration rose during un-induced cultivation at constant feeding (Figure 3). Consistent with the observation that batch product formation rates were highest when cells were induced at μ_max_, the induction in fed-batch during fast feeding (growth rate higher) led to a higher accumulation of DAMP4 and process time was shorter than at slow feeding. This can be explained by previous observations that *E. coli* exhibits both a more efficient amino acid synthesis and charging of tRNAs [51] and a more efficient energy generation [52] during recombinant protein production at fast feeding. The undesired accumulation of acetate [53] in the fed-batch experiments was moderate. A maximum of around 4 g/L could be observed, which was also consistent with the batch data. This means that in the case of DAMP4 production with BL21(DE3), we did not reach the inhibitory concentration of 5 g/L reported previously [20]. In some cases acetate remained below 1 g/L and most certainly, the ceasing of DAMP4 accumulation was not caused by acetate toxicity; indeed most acetate accumulated after peptide production stopped (Figure 3).

## Conclusion

In this work we established the production of the short surfactant peptide DAMP4 on chemically defined medium, extended production to sucrose by re-engineering the existing production process to metabolize sucrose and increased product titer to several grams per liter of culture through the use of high cell density cultures (HCDC). Significantly, expression of DAMP4 was stable and product titer was similar on glucose or sucrose. Data showed that more than 6.5 g/L of the recombinant peptide could be produced from both glucose and sucrose. The production process reported in this work has model characteristics for the efficient production of other designed peptides.

## Materials and methods

### Bacterial strains and plasmids

*E. coli* BL21(DE3) was the expression host. The peptide surfactant DAMP4 NH_2_-MD(PSMKQLADS-LHQLARQ-VSRLEHAD)_4_-COOH was expressed from the pET48b(+) (Merck Millipore, Kilsyth, Victoria, Australia) based vector pEDA [15]. The plasmid was maintained using Kanamycin (Merck Millipore) at 50 μg/ml final concentration. Expression of DAMP4 was induced by the addition of IPTG (Merck Millipore) at 1 mM final concentration. Sucrose catabolism was conferred by plasmid p15aCSCx, carrying the *cscAKB* operon from *E. coli* W [31] expressed from a pACYC184 [54] based vector. Selection was achieved using sucrose. Transformations were performed as described in [25]. The resulting strains *E. coli* BL21(DE3) pEDA and *E. coli* BL21(DE3) pEDA,p15aCSCx were stored in 50% glycerol-R/2 solution at −80°C. DAMP4 gene was sequence-verified before and after cultivation.

### Media and chemicals

All chemicals were of analytical grade and obtained from Sigma Aldrich (Castle Hill, NSW, Australia). Ultrapure water (18 MΩ/cm) was used. Tryptone, yeast extract and agar were obtained from Becton, Dickinson & Co., Sparks, MD, USA.

*Complex medium*: Lysogenic broth (LB) [55] with 20 g/L glucose (LBG) was the liquid complex medium. Solid medium contained 15 g/L agar. All media were sterilized by autoclaving, except for glucose solution that was filter sterilized and added separately.

*Chemically defined minimal medium (CDM)* [56]: R/2 was adjusted to pH 6.9 with KOH and supplemented with either sucrose (CDM_S_) or glucose (CDM_G_) as the sole carbon source at a final concentration of 20 g/L. CDM was filter sterilized with a 0.22 μm polyethersulfone membrane (Merck Millipore).

### Determination of cell density

Cell growth was followed by optical density at 600nm (OD_600_) in a UV–VIS spectrophotometer (Libra S4, Cambridge, England). For LBG medium, blanks were performed against LBG medium in the same aqueous dilution as the sample. For CDM, water was used as blank. Dry cell weight was determined collecting 2 mL of culture medium in triplicate in a pre-weighted tube, which was centrifuged (3K30, Sigma Laborzentrifugen Gmbh, Osterode, Germany) at 13,500 × g, 4°C for 5 min, the cell pellet was then collected and washed with ice-cold water, re-pelleted, frozen and freeze-dried until constant weight was obtained.

### Cultivation conditions

All cultivations were performed at least in biological duplicates. Bioreactor experiments were carried out in parallel bioreactor system (DasGip, Juelich, Germany). The pH was maintained at 6.9 by addition of 25% NH_4_OH. Dissolved Oxygen (DO) was monitored by an external DO meter (Presens, Regensburg, Germany) and maintained at 70% of air saturation by automatic control of pure O_2_ enrichment. Temperature, stirring and gas flow were kept at 37°C, 800 rpm and 12 standard liters per hour (sL/h) using electronic mass flow control, respectively. Concentration of CO_2_ in the off-gas was monitored online using a gas analyzer (GA04, DasGip). Reactor volume, gas flow and CO_2_ concentrations were used to determine the CTR and hence the amount of CO_2_ (mmol-CO_2_) transferred during cultivation. Initial culture volume was 0.2 L, containing 0.1% v/v Antifoam C. Inocula for bioreactors were generated in shake flasks (250 mL baffled flasks in a Multitron orbital (2.5 cm) shaker (Infors, Noble Park North, VIC, Australia) at 200 rpm, 37°C and 50 mL culture volume), as described before [25] using the same medium as the respective main culture. Experimental data were used to calculate the cell density for induction to consume a given quantity of substrate using the formula DCW_i_ = SS_total_/(Y_xs_ + Y’_xs_), where DCW_i_ is the dry cell weight at the point of induction [g/L], SS_total_ is the total substrate concentration [g/L], Y_xs_ and Y’_xs_ are the amount of sugar needed per unit of biomass generated before and after induction, respectively [g/g]. Fed-batch cultivations started as a batch culture and constant feeding commenced at ~9g/L DCW, approximately 5 hours after culture start. The feeding regimen was 5.5 mL/h for fast feeding (feed rate = F_F_) and 4.6 mL/h for slow feeding (feed rate = F_S_); the feed consisted of carbon source (600g/L), MgSO_4_ (10g/L), (NH_4_)_2_SO_4_ (10g/L), kanamycin (50 μg/ml), Antifoam C (0.1% v/v) and water. Feed rate was calculated based on the desired cell density and growth rate at induction as reported in [45].

### *Quantification of substrates and products*

Samples for extracellular metabolite analysis were collected at regular intervals during the entire process and samples for protein analysis were collected immediately prior to induction and at regular intervals afterwards. For each sample point, cells were harvested by centrifugation (13,500 × g, 4°C, 5 min). OD_600_ was recorded, the supernatant was transferred to a fresh tube and the cell pellet was washed with sterile H_2_O; both samples were kept at −80°C until further use. Prior to HPLC injection, extracellular metabolite samples were thawed on ice and filtered with a 3 KDa molecular weight cut-off filter (Merck Millipore) according to manufacturer’s directions. HPLC analysis was performed as described previously [25]. The substrates and products quantified by HPLC were formate, acetate, and glucose, fructose and sucrose.

DAMP4 was quantified using SDS-PAGE. For this, cell pellets were resuspended in 10% solution BugBuster 10X reagent (Merck Millipore) and sterile H_2_O, such that the total protein concentration of the final mixture was suitable for loading on the gels. An aliquot was withdrawn and mixed (1:2) with Laemmli Sample Buffer (Biorad, Gladesville, NSW, Australia) and incubated (12 min, 80°C). Identical protein amounts (1–0.1 μg/well) were loaded onto precast 12% NuPAGE SDS-PAGE gels (Life Technologies, Mulgrave, Victoria, Australia) and run for 65 min at 200V in MES buffer (Life Technologies). Gels were rinsed 3 times with H_2_O, stained with Coomassie Blue (Life Technologies) for 30 min, destained in H_2_O overnight, imaged and analyzed with Image Lab 4.0 (Biorad, Gladesville, NSW, Australia) using 3 mm background correction sphere. Quantification was then achieved by densitometry using a standard curve (0.75, 0.5, 0.25, 0.1 μg/well) of purified DAMP4 (purity > 90% by HPLC) included on each gel and by determining the abundance of recombinant DAMP4 on total cell proteins and expressed as % of total cell protein (D4/TCP). About ~92 ± 10% of the spiked pure peptide on a negative control could be recovered. Total cell protein (TCP) was determined using Bradford assay (Thermo Fisher Scientific Australia, Scoresby Victoria, Australia). DAMP4 was not detected in supernatant fractions.

## Additional files

Additional file 1: Figure S1. Growth profiles, product accumulation and extracellular metabolite profiles for batch cultivation in chemically defined medium with glucose or sucrose as sole carbon source for induction performed at DCW_l_ and DCW_h_. Additional file 2: Table S1. Yields of recombinant DAMP4 using either Sucrose or Glucose substrates and induced at DCW_l_ or DCW_h_ during batch cultivation or induced at DCWi with Fs feeding regimen for fed-batch cultivation. **Table S2.** Rates of consumption/production for main cultivation parameters in Batch or Fed-batch cultivation. Additional file 3: Figure S2. Growth profiles, product accumulation and extracellular metabolite profiles for fed-batch cultivation in chemically defined medium with glucose or sucrose as sole carbon source for induction performed at DCWi ~ 40g/L and Fs feeding regimen.
