# Supplementary figures and images for: Production of the short peptide surfactant DAMP4 from glucose or sucrose in high cell density cultures of Escherichia coli BL21(DE3)
Source: Microb Cell Fact. 2014 Aug 19;13:99. doi: 10.1186/s12934-014-0099-y (PMC4229601; doi:10.1186/s12934-014-0099-y)

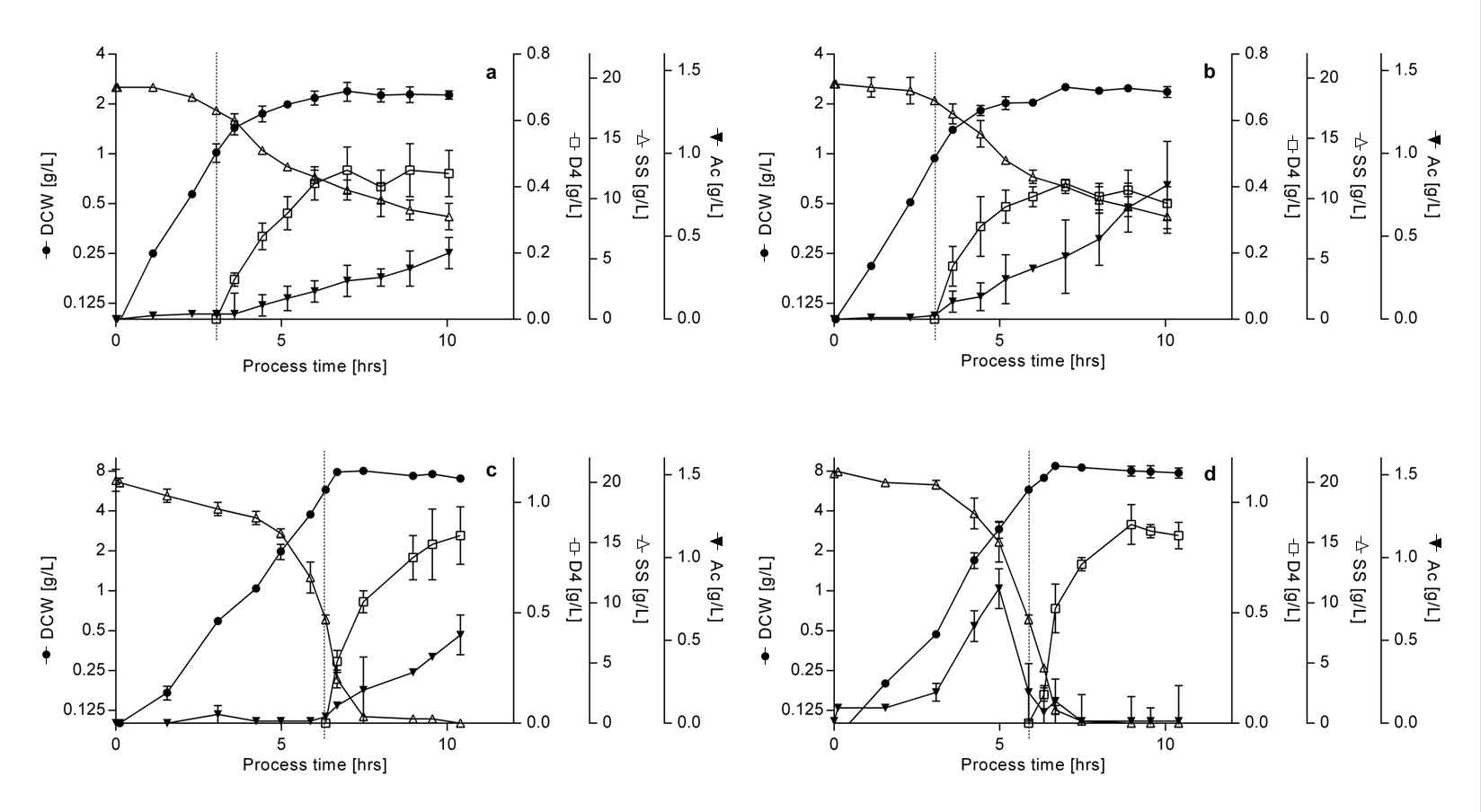

Supplement: Additional file 1: Figure S1. — Growth profiles, product accumulation and extracellular metabolite profiles for batch cultivation in chemically defined medium with glucose or sucrose as sole carbon source for induction performed at DCWl and DCWh. [file 12934_2014_99_MOESM1_ESM.tiff]

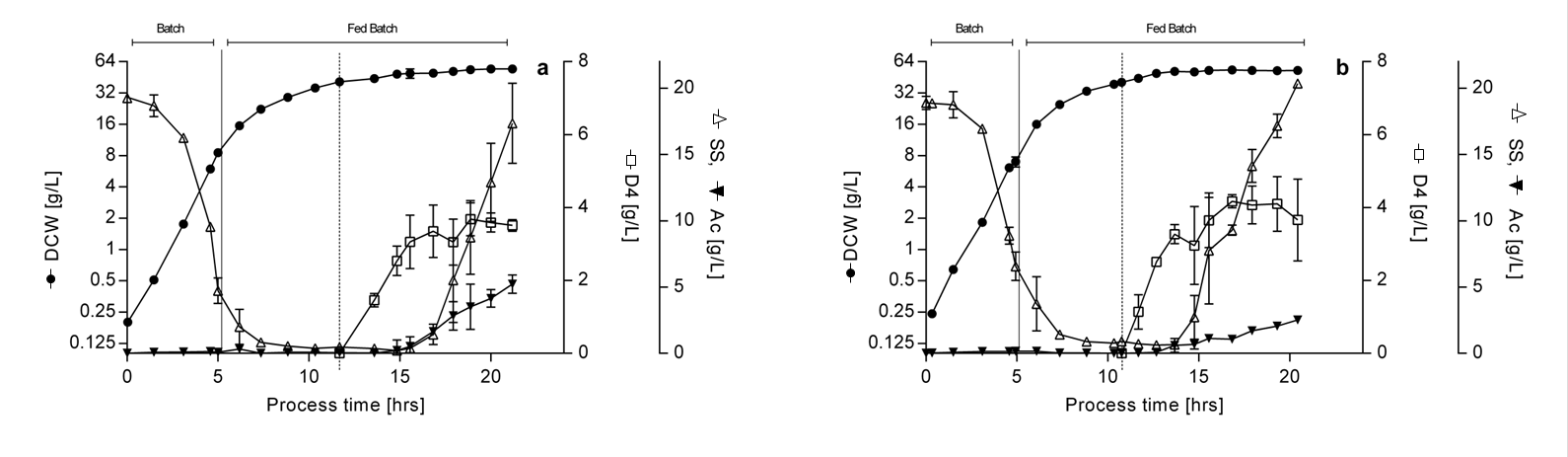

Supplement: Additional file 3: Figure S2. — Growth profiles, product accumulation and extracellular metabolite profiles for fed-batch cultivation in chemically defined medium with glucose or sucrose as sole carbon source for induction performed at DCWi ~ 40g/L and Fs feeding regimen. [file 12934_2014_99_MOESM3_ESM.tiff]
