# Supplementary material for: Production of the short peptide surfactant DAMP4 from glucose or sucrose in high cell density cultures of Escherichia coli BL21(DE3)
Source: Microb Cell Fact. 2014 Aug 19;13:99. doi: 10.1186/s12934-014-0099-y (PMC4229601; doi:10.1186/s12934-014-0099-y)
Supplement: Additional file 2: Table S1. — Yields of recombinant DAMP4 using either Sucrose or Glucose substrates and induced at DCWl or DCWh during batch cultivation or induced at DCWi with Fs feeding regimen for fed-batch cultivation. Table S2. Rates of consumption/production for main cultivation parameters in Batch or Fed-batch cultivation. [file 12934_2014_99_MOESM2_ESM.pdf]

Supplementary Material

Production of the short peptide surfactant DAMP4 from glucose or sucrose in high cell density cultures of *Escherichia coli* BL21(DE3)

Michele Bruschi[a], Jens O. Krömer[b*], Jennifer A. Steen[a], Lars K. Nielsen[a]

Supplementary text:

▪ *Batch cultivation*

To assess the impact of cell density at induction on DAMP4 accumulation, production in batch operations was characterized inducing at different cell densities. Induction started product accumulation, decreasing growth rate until cell division and production halted acetate accumulation commenced as well (Figure 1s). Induction at low biomass concentration (DCW_l) yielded ~2.4 g/L DCW and ~0.4 g/L DAMP4 (36% DAMP4/TCP) in a ~7 h process for glucose and sucrose, respectively. As observed in the main text, product accumulation was not detected during stationary phase or in the extracellular space. At harvest, about half of the substrate supplied had not been metabolized (Figure 1s a, b). Performing induction at high biomass concentration (DCW_h, Figure 1s c, d) more than 7 g/L DCW for both substrates was obtained and product titer was ~0.9 g/L, corresponding to ~26% DAMP4/TCP. Using this strategy, sugar completely depleted during production, which reduced specific productivity because of the insufficient substrate availability for product generation. Collected data indicated that ~10g of sugar are necessary per g of DAMP4; to ensure maximum specific productivity, therefore, cells should be supplied with adequate substrate. Fed-batch cultivation overcame this requirement.

▪*Fed-batch cultivation*

To assess the impact of growth rate before induction (i.e.: during feed phase) on product formation, fed-batch cultivation was performed supplying limiting substrate in a linear fashion at two different

feeding regimen. Induction was performed at constant DCW. Average biomass formation rate during production phase dropped to ~0.05 h^{-1} for all conditions regardless of the feeding conditions, in contrast to the feeding phase where growth rate was different for different feeding regimens. More than 50 g/L final cell density was obtained for all cultures. As discussed in the main text, maximum product accumulation was reached when growth rate before induction was high (0.33 h^{-1}, F_f feed), whereas lowering growth rate before induction (~0.29 h^{-1}, F_s feed) maximum product titer reached ~4.7g/L (~18% DAMP4/TCP) on sucrose, ~60% lower than for the other feeding regimen (Figure 2s). Acetate was produced mainly after induction.

▪ *Yields and rates*

Yields of product during the whole cultivation period and over the production phase are reported in Table 1s. Product formation was more efficient for induction at high cell density, suggesting that production is more efficient at early induction phase. Table 2s reports the rates for fed-batch cultivations. Rates were similar for all the conditions tested, except for growth during feeding and product formation rate.

Supplementary tables:

Table 1s: Yields of recombinant DAMP4 using either Sucrose or Glucose substrates (*Substrate*) and induced at DCW_l or DCW_h during batch (*Batch*) cultivation or induced at DCW_i with F_s feeding regimen for fed-batch cultivation (*Fed-Batch, Induction point*, *Feeding rate*). Specific productivity ($Y_{D4/DCW}$) and substrate conversion into product ($Y_{D4/ss}$) composed of the yield for the whole process (*Total*) and for the sole production phase (*Production*) are shown. Experimental error represented as SD, n≥2.

	Substrate	Induction point, Feeding rate [g/L, mL/h]	$Y_{D4/DCW}$ [g/g]	$Y_{D4/ss}$ [%] Total	Production
▪ **Batch**					
G_l	Glucose	1, NA	195.48±8.36	6.7±0.6	8.8±1.0
S_l	Sucrose	1, NA	162.44±8.44	6.5±0.2	8.5±0.8
G_h	Glucose	6, NA	121.07±9.35	5.4±0.6	12.3±1.2
S_h	Sucrose	6, NA	110.10±8.65	5.2±0.8	11.5±1.4
▪ **Fed-Batch**					
G_s	Glucose	40, 4.3	83.44±9.66	3.0±0.3	6.0±0.7
S_s	Sucrose	40, 4.3	76.68±5.64	3.6±0.1	6.7±1.3

Table 2s: Rates of consumption/production for main cultivation parameters in Batch or Fed-batch cultivation expressed as [mmol-C/gDCW*h] unless otherwise stated. *Name* is the experiment identifier; *Substrate* denotes whether glucose or sucrose was the substrate; *Phase* connotes batch Growth phase (*G*), growth during Feeding (*F*, when applicable) and Production (*P*) phases, which is defined as the period between induction and the peak of product accumulation. *GR*, indicates Growth rate and *SUR*, Substrate Uptake Rate; *CO_2*, *Ac*, *Fo* and D4 denote CO_2, Acetate, Formate and recombinant DAMP4 production rate respectively; *Balance* is the percentage ratio between substrate consumption and product formation rates. ND: Not Determined. Experimental error expressed as SD, standard deviation, n≥2.

**Name**	**Substrate**	**Phase**	**SUR**	**DCW**	**CO_2**	**Ac**	**Fo**	**D4**	**Balance [%]**
▪ **Batch**									
G_l	Glucose	G	53.0 ± 2.0	24.8 ± 2.1	24.0 ± 1.8	1.5 ± 0.1	1.4 ± 0.2	0.0 ± 0.0	102.5
		P	24.7 ± 1.3	8.2 ± 0.4	13.2 ± 1.6	2.2 ± 0.1	0.0 ± 0.0	2.2 ± 0.1	95.5
S_l	Sucrose	G	53.6 ± 1.2	23.1 ± 1.7	23.8 ± 2.9	1.1 ± 0.2	3.2 ± 0.2	0.0 ± 0.0	104.5
		P	28.8 ± 1.0	9.5 ± 0.8	12.2 ± 0.6	2.2 ± 0.1	0.0 ± 0.1	2.4 ± 0.2	109.4
G_h	Glucose	G	46.4 ± 0.5	22.7 ± 0.6	19.4 ± 1.3	1.3 ± 0.1	0.8 ± 0.1	0.0 ± 0.0	104.8
		P	24.6 ± 0.2	9.2 ± 0.2	12.1 ± 0.1	1.7 ± 0.1	0.0 ± 0.0	2.3 ± 0.1	97.3
S_h	Sucrose	G	52.0 ± 0.4	24.0 ± 0.7	22.0 ± 0.1	1.4 ± 0.1	2.1 ± 0.1	0.0 ± 0.0	105.1
		P	22.2 ± 0.6	7.7 ± 0.5	11.2 ± 0.2	1.2 ± 0.0	0.0 ± 0.0	2.2 ± 0.1	99.4
▪ **Fed Batch**									
G_s	Glucose	G	56.9 ± 1.1	23.9 ± 0.2	26.3 ± 1.0	0.6 ± 0.1	1.0 ± 0.2	0.0 ± 0.0	109.5
		F	18.0 ± 0.7	7.8 ± 0.2	10.4 ± 0.1	0.0 ± 0.0	0.1 ± 0.0	0.0 ± 0.0	98.5
		P	4.6 ± 1.0	1.5 ± 0.3	3.1 ± 0.6	0.1 ± 0.0	0.0 ± 0.0	0.2 ± 0.0	94.8
S_s	Sucrose	G	56.3 ± 1.3	23.7 ± 0.6	27.1 ± 2.1	0.7 ± 0.2	1.3 ± 0.2	0.0 ± 0.0	106.5
		F	22.9 ± 1.3	8.8 ± 0.6	12.1 ± 0.6	0.0 ± 0.0	0.2 ± 0.1	0.0 ± 0.0	108.8
		P	5.4 ± 0.6	1.6 ± 0.1	3.6 ± 0.1	0.1 ± 0.0	0.0 ± 0.0	0.3 ± 0.0	96.6

Supplementary figures:

Figure 1s: growth profiles, product accumulation and extracellular metabolite profiles for batch cultivation in chemically defined medium with glucose (left) or sucrose (right) as sole carbon source for induction performed at DCW_l and DCW_h as indicated by the arrow. Biomass formation (*DCW*, circles) is reported on a logarithmic scale; product accumulation (*DAMP4*, open squares), substrate concentration (*SS*, open triangles) and acetate accumulation (*Ac*, inverted filled triangles) are shown. Experimental error represented as SD, $n \geq 2$.

Figure 2s: growth profiles, product accumulation and extracellular metabolite profiles for fed-batch cultivation in chemically defined medium with glucose (left) or sucrose (right) as sole carbon source for induction performed at DCW_i ~ 40g/L as indicated by the arrow and F_s feeding regimen. Biomass formation (*DCW*, circles) is reported on a logarithmic scale; product accumulation (*DAMP4*, open squares), substrate concentration (*SS*, open triangles) and acetate accumulation (*Ac*, inverted filled triangles) are shown. Experimental error represented as SD, $n \geq 2$.
